# Ethical and cultural implications for conducting verbal autopsies in South and Southeast Asia: a qualitative study

**DOI:** 10.1136/bmjgh-2023-013462

**Published:** 2023-12-11

**Authors:** Nan Shwe Nwe Htun, Carlo Perrone, Aung Pyae Phyo, Aninda Sen, Koukeo Phommasone, Moul Vanna, Nipaphan Kanthawang, Jarntrah Sappayabanphot, Widi Yotyingaphiram, Jindaporn Wirachonphaophong, Nawrin Kabir, Sam Ol, Xaipasong Xaiyaphet, Ailatda Soulivong, Khambang Seevanhthong, Rupam Tripura, Rusheng Chew, Napat Khirikoekkong, Shaun K Morris, Anne Osterrieder, Phaik Yeong Cheah, Prabhat Jha, Yoel Lubell, Thomas J Peto

**Affiliations:** 1 Mahidol-Oxford Tropical Medicine Research Unit, Faculty of Tropical Medicine, Mahidol University, Bangkok, Thailand; 2 Centre for Tropical Medicine and Global Health, Nuffield Department of Medicine, University of Oxford, Oxford, UK; 3 Shoklo Malaria Research Unit, Mae Sod, Thailand; 4 Communicable Diseases Programme, BRAC, Dhaka, Dhaka District, Bangladesh; 5 Lao-Oxford-Mahosot Hospital-Wellcome Trust Research Unit, Vientiane, Lao People's Democratic Republic; 6 Action for Health Development, Battambang, Cambodia; 7 Division of Infectious Diseases, Child Health Evaluation Sciences and Centre for Global Child Health, Hospital for Sick Children, Toronto, Toronto, ON M5G 1E8, Canada; 8 University of Toronto Dalla Lana School of Public Health, Toronto, Toronto, ON M5T 3M7, Canada; 9 Centre for Global Health Research, St.Michael's Hospital, Toronto, Ontario, Canada

**Keywords:** Qualitative study, Epidemiology, Public Health

## Abstract

**Introduction:**

Causes of deaths often go unrecorded in lower income countries, yet this information is critical. Verbal autopsy is a questionnaire interview with a family member or caregiver to elicit the symptoms and circumstances preceding a death and assign a probable cause. The social and cultural aspects of verbal autopsy have gotten less attention than the technical aspects and have not been widely explored in South and Southeast Asia settings.

**Methods:**

Between October 2021 and March 2023, prior to implementing a verbal autopsy study at rural sites in Bangladesh, Cambodia, Laos, Myanmar and Thailand, focus group discussions were conducted with village heads, religious leaders and community members from varied demographic backgrounds. Thematic analysis elucidated customs and traditional views surrounding death to understand local ethnocultural sensitivities.

**Results:**

We found that death rituals varied greatly among religions, ethnicities and by socioeconomic status. Mourning periods were reported to last 3–100 days and related to the cause of death, age and how close the deceased person was to the family. Participants advised that interviews should happen after mourning periods to avoid emotional distress, but not long after so as to avoid recall bias. Interviewers should be introduced to respondents by a trusted local person. To provide reassurance and confidentiality, a family’s residence is the preferred interview location. Interview questions require careful local language translation, and community sensitisation is important before data collection.

**Conclusion:**

Verbal autopsy is acceptable across a wide range of cultural settings in Southeast Asia, provided that local norms are preidentified and followed.

**WHAT IS ALREADY KNOWN ON THIS TOPIC**

- The verbal autopsy (VA) method can be sensitive to the individuals interviewed, their families and communities. Ethical, social and cultural issues in VA have received less attention than the technical aspects and have not been widely explored in South and Southeast Asia.

**WHAT THIS STUDY ADDS**

- We found that VA is acceptable across a wide range of cultural settings in rural South and Southeast Asia, provided that local norms are preidentified and followed.

**HOW THIS STUDY MIGHT AFFECT RESEARCH, PRACTICE OR POLICY**

- The local cultural traditions and sensitivities towards VA procedures we have identified in this study provide guidance to interview teams in other such studies as to the issues to be taken into consideration and how these can be approached in advance and, therefore, the focus group discussion’s findings will enrich the literature on the VA research.

## Introduction

In low and middle-income countries (LMICs), mortality statistics are commonly limited by poor administrative systems, insufficient resources and low coverage of death notifications due to a lack of legal enforcement.^1 2^ The majority of people still die at home, without contact with the formal healthcare system, and the causes of death are largely unknown.^3^ Mortality data are vital to helping formulate public health policy, allocate medical resources efficiently and evaluate public healthcare programmes.^4^ The civil and vital registration system in the majority of South and Southeast Asian countries are still insufficient, which particularly affects poor and rural communities. To fill these information gaps, verbal autopsy (VA) has been a practicable approach in some countries of the region (India, Indonesia, Thailand, Malaysia, Vietnam and Bangladesh) in the recent years,^5^ to determine the most likely causes of death by interviewing a family member or caregiver to gather information about the symptoms and circumstances of the deceased person in the period leading up to their death.^6 7^

Obtaining information on causes of death can be sensitive to the individuals interviewed, their families and communities.^8 9^ Several studies have addressed the design and content of the questionnaires, interview procedures and research skills of the interviewers, which impact the validity, reliability and study outcomes.^10^ Ethical, social and cultural issues in VA have received less attention than the technical aspects in the region and elsewhere, yet adherence to local cultural practices and engagement with communities throughout the study process is crucial for successful implementation of VA.^11^

The current focus of the South and Southeast Asia Community-based Trial Network (SEACTN) research programme is the epidemiological baseline Rural Febrile Illness (RFI) project, in approximately 520 villages across five South and Southeast Asian countries (Bangladesh, Cambodia, Lao PDR, Myanmar and Thailand), capturing over 100 000 episodes and outcomes of fever in these remote and underserved communities.^12^ Mortality statistics in the populations covered by SEACTN are considered as generally limited and unreliable and, therefore, in parallel, verbal autopsies are carried out to identify not only potential causes of death with a history of febrile illnesses but also all other causes of death.^12^ As VAs have not been used previously at the study sites, prior to its implementation, we sought to explore local beliefs and practices about death to inform appropriate bioethical practices.

## Methods

**Figure 1:**
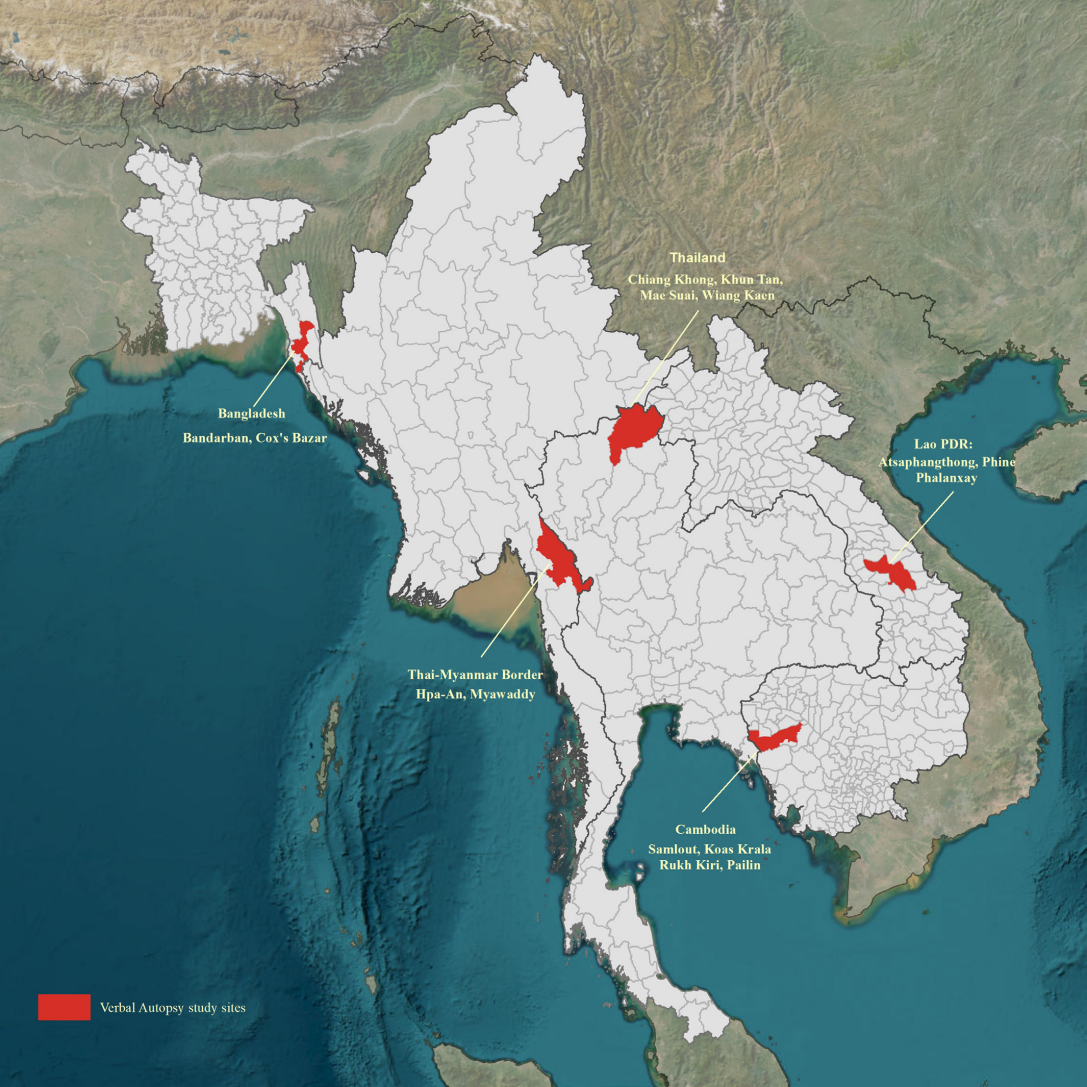
Verbal autopsy study countries and sites, where the focus group discussions took place.

The research design was a qualitative study with face-to-face focus group discussion (FGD) approach before the implementation of a VA study (NCT04595656). FGDs were conducted at seven locations in five different countries (Cox’s Bazar and Bandarban districts on the southeast coast of Bangladesh; Chiang Khong, Khun Tan, Mae Suai and Wiang Kaen districts of Chiang Rai province in northern Thailand; Hpa-An and Myawaddy districts of Karen state on the border between Myanmar and Thailand; Atsaphangthong, Phine and Phalanxay districts of Savannakhet province in southern Laos; Samlout, Koas Krala and Rukh Kiri districts of Battambang and Pailin provinces in the far northwest of Cambodia (figure 1). In Chiang Rai, northern Thailand and on the Thai-Myanmar border, there are multiple ethnic groups who have specific local traditions and beliefs. Thus, an additional FGD was carried out at these sites to capture their different views and opinions. The selected areas of the study countries are rural areas, frequently characterised by high levels of poverty; limited access to healthcare; a lack of information on the causes of disease and death in these areas, leading to difficult to determine which interventions should be scaled up.^12^ These study areas were chosen because they are part of the SEACTN programme (detailed above) and these are all areas participating in a major febrile illness aetiology study, and deaths within all participating villages were included in this VA study, and, therefore, no further sampling frame was adopted. The FGDs were done between October 2021 and March 2023, staggered due to the evolving COVID-19 pandemic situation in the region. The focus group participants were purposefully sampled to ensure diverse demographic backgrounds of age, gender and ethnicities and they were broadly representative to the study population to record their different points of views. The selected participants were first informed about the study protocols and activity, and when they were willing to participate, they were advised of the time and central location of the activity. The participants received a daily allowance, which covered their transportation reimbursements and a meal, in exchange for their participation, and we followed the established ethical guideline of the local study team.

Researchers with knowledge of the local context and fluency in the local language conducted FGDs at each site. Guidelines and key themes for FGD were developed from the literature and the country’s social factors. Individual written informed consent was obtained from all participants. With an interview guideline, facilitators commenced the participants by asking broader questions about the topic of interest (customs and perceptions about deaths), before asking the specific questions on VA study. Even though each participant responded to the facilitator’s questions on their own, they were encouraged to converse and connect with one another by exploring and elaborating on their common and different viewpoints. Each FGD lasted about 2 hours. Participants were not identified by name in the audio recordings or field notes. The audio recordings were transcribed and translated to English. After revisiting the research questions and aims, we analysed the data by thematic analysis using NVivo V.10. The contents were classified in accordance with the various themes, and if the data within the theme revealed any subthemes that were noteworthy or important to the questions we were trying to answer, the subthemes were then identified. We reviewed all the themes and subthemes and made sure they were accurate and comprehensive and also to check for any themes that were left off. The themes were finally labelled, finalised and described in detail. Then, the findings were shared with local study teams and did consultation meetings to clarify the contents and to ensure the correct interpretation. We also adhered to the guideline for authors’ reflexibility statement to promote equitable authorship in the publication of research from international partnerships (online supplemental appendix 2).

10.1136/bmjgh-2023-013462.supp2 Supplementary data

### Patient and public involvement

Neither patients, parents nor the public were involved in the design, conduct or reporting of this research.

## Results

A total of 70 participants were involved in the FGDs. Participant characteristics are presented in table 1. The median age of participants was 43 (IQR 31–54), and 35/70 (50%) were women. Professionally, the participants were from both the public and private sectors and play an important role in their communities. Beliefs and practices about death and opinions about VA study-related procedures were discussed, and the findings from all FGDs are presented by theme. The main themes articulated by study participants included (1) death rituals among different religions and ethnicities; (2) how the VA interview process should be done and (3) ethnocultural factors at each study site. Each theme has a number of subthemes, which are presented in brief. The detailed responses from the FGDs are also described separately by country online supplemental appendix 1).

10.1136/bmjgh-2023-013462.supp1 Supplementary data

**Table 1:** Characteristics of focus group participants

Study countries	Role of FGD participants in the community	Gender		Total
		Male	Female	
Bangladesh	Buddhist monk, university student, headmaster, housewife, businessman/woman, healthcare worker, retired government officer, community healthcare providers, representative of community-based women activist group Ethnic group: Marma	6	6	12
Cambodia	Village chiefs, representative from community woman affair network, health centre staff, retired government staff, primary school committee member, staff from community forestry association, archbishop’s committee member The majority were Khmers.	7	5	12
Lao PDR	Farmers, deputy village heads, livelihoods The majority were Laos.	6	3	9
Thai-Myanmar border	FGD1: village track leaders, vendor, medical staff, village track administrative staff, midwife, social volunteer, community health worker, teacher, business woman, religious leader Ethnic groups: S’gaw-Karen, Pwo-Karen, Burmans/Bamars	6	8	14
	FGD2: village committee member, teachers, village health workers, representative from women organisation and religious leaders Ethnic group: S’gaw Karen	4	4	8
Thailand	FGD1—farmer, deputy village head, vendor, police inspector, mayor’s secretary, gardener, agriculturists Ethic groups: Tai Yai (Shan), Yunnan Chinese, Khmu, Hmong and Lao-Thai	4	4	8
	FGD2—farmers, religious leader, village health volunteers Ethnic groups: Lisu, Akha, Lahu	2	5	7

FGD, focus group discussion.

### Death rituals and perceptions about deaths

The ritual practices vary among religions, and ethnicities and are also dependent on available resources. The majority of the population in Laos, Thailand, Thai-Myanmar border area and Cambodia are Buddhists, and they share similar principles. In Buddhism, when a person dies, the body is immediately washed, dressed, placed into a coffin and kept in the house for 3 to 7 days before cremation or burial. The monks are invited home or the body is brought to the monastery and a sermon or to chant are recited for blessings. After the body is cremated, the ashes are collected to be spread into the water or, if buried, a small house archway with a pagoda or stupa is made within the temple or cemetery compound. If the deceased is a Christian, they are normally buried, and religious leaders are invited to pray and read the Bible at the ceremony. Then, the family holds the funeral ceremony and mourns, for a period of time that depends on their socioeconomic status. As a Cambodian participant described,

In the Khmer community (both Buddhists and Christians), the rich ones can hold the ceremony for a longer period (many days, even months, like a 100-day anniversary or yearly anniversary), while the poor ones are only able to have it for as short as 1–3 days.

In general, when the village head announces someone’s death (the Laotians put a notification in front of the deceased’s house for 3 days), the community reaches out to the deceased’s family to help cook food items that are shared to host the guests, and also provide emotional support and financial contributions.

In the Muslim community of Bangladesh, the body is first bathed with water, placed on a bamboo platform called ‘pati’ in Bengali or wooden coffin (called ‘khatia’), and then covered nicely with pieces of clothing. Relatives, neighbours and religious leaders pray for the deceased at home, or at the mosque or at the graveyard and the body is buried. One Muslim participant remarked,

When there is a death, just after that, burial should be, and the sooner the burial activities are completed, the more virtue it will carry.

In Bangladesh, adults who follow Buddhism or Hinduism either bury or cremate the body, depending on the deceased’s wishes. However, Hindu children under 12 years old and Buddhist children are usually buried. Between the 5th and 7th day, underprivileged people are fed by the family, believing that doing so, the deceased will receive more virtue in the afterlife. This is also done after 1 month and after 1 year, according to the economic capabilities of the family.

Representatives contemplated death, accepting it as a fact of human life. One Lao said,

Being humans, we all die one day, especially the elders, whom we can consider ripe fruits that naturally drop. We are born, become old, become ill, and die, which everyone understands.

The FGD informed that it would be possible to conduct VA interview if we identify local norms. However, the group participants across the sites noted that the cause of death mattered when the death of their beloved relatives was discussed in their communities. One Thai participant described,

It might depend on case by case, and what is the cause of death, for example, a transmission disease like HIV or being killed by someone, the family might have a hard time because it’s something that they can’t tell or don’t want to talk about, but if it’s something general like an elderly disease, they can talk about it, and I don’t think it’s a problem.

Similarly, one Bangladeshi shared that there might be some difficulties in having a conversation with the community if the death was unnatural, such as hanging, suicidal poisoning or any mysterious case, as people would not feel comfortable talking about such issues, or sometimes they might try to avoid them when the police were involved. This may prevent or obstruct gathering of information.

### The VA interview process

#### How to establish trust to increase community participation?

In our study settings, when a villager dies, the village authorities and health centre staff get notified by the villagers, which is a very common death-informing pathway. These stakeholders are influential in managing social and health-related issues in their villages. The study was supported by the participants during the public engagement activities. Building trust between the research team and the community is critical for research implementation.

A Thai respondent expressed,

As the first thing, ask the leader to introduce the interviewers to us, explaining that the researchers will come and interview about death. If someone from the community, either the leader or primary care unit or, municipality staff (tesaban), or village health volunteers, goes into the community, we are familiar with their faces. We will trust and provide the information if we are asked extensive questions.

Other village representatives also perceived that it was better if they accompanied the research team or interviewers, as the community usually liked to be visited by them, because the families would feel supported. One Lao village representative said,

On the day of death, the village authorities usually hand over the tribute fund to the family, and we will be able to inquire about the interview and help coordinate the visit for you. If you visit or interview the families alone, they may not cooperate, I can assure you.

This scenario was agreed on by the respondents at all sites. The village authorities or village healthcare providers are recognised as insiders in the community who could usually connect the deceased families and the research team, get approval, schedule an appointment to be made and define the interview location.

In addition, community meetings were necessary to introduce the project to the communities in order to avoid potential barriers. One S’gaw Karen stated,

This study, in my opinion, would not be problematic at all and this would help us to understand more. As I can tell, some families have less knowledge and understanding, it would be beneficial to hold a community meeting with the leaders and villagers and clearly explain the project to them as well as the reason why there would be an interview with the families asking about the death. We live in the same village; we have to bond and we have to cooperate with the village, I believe.

#### When to conduct the interview?

Mourning periods greatly differ across the study sites and are dependent on religions, cultures, the cause of death, the age of the deceased and how close the family was to the deceased. In general, this ranges from 3 days to around 100 days. Typically for Hindus, it ranges from 7 to 40 days; for Buddhists, it is mostly 7 days, 4 days up to 6 months for Muslims and 3–40 days for Christians. Some practices by other ethnic groups are mentioned in detail in online supplemental appendix 1.

Participants suggested that visiting families to carry out the interviews could be done soon after the funeral ceremony (usually after 7 days), but not before the funeral, as the people are still in a grieving stage and rituals are performed during that time. Communities in Thailand (including Khmu, Tai Yai and Lao ethnicities) expressed,

In our community, we mourn about one week after death and rituals are performed at that time and the families are usually busy. After a week, the interview can be done with the families.

Mourning for elderly people or a person with a known disease status usually lasts for 1 week to 1 month, but mourning for those with accidental deaths is more serious and prolonged due to the sudden onset, and families often do not cope well with the situation at the beginning. One S’gaw Karen stated,

My mother-in-law passed away due to her advanced age. She has been ill and endured a lot due to her illnesses and we have great sympathy for her. We grieved for a few days after her death and accepted it as part of life. In our village, when a young child died after falling from a tree, we are still talking about it and grieving over it even after one year. Imagine the feelings of his family!

Moreover, the mourning period takes longer if the deceased is a child. If the respondent is a mother, she would need more time to stabilise her emotions, and in such cases, the interview should be delayed, perhaps not done before 2–3 months. A Bangladeshi mother expressed,

I really mourned for a long time when my first child died. I missed him a lot. I only began to recover after my second child was born.

However, some participants emphasised that the interviews should not be conducted more than a few months after someone has passed away, otherwise, the family would not remember the details, and the village chief would also not remember who died or when. The majority of the participants shared that after completing the religious rituals surrounding someone’s death, people resumed working, as most people in these communities live on their daily wages or the adults were mostly occupied in the agricultural fields during harvesting seasons or a primary caregiver who had already left the village after the deceased passed away. These are the usual circumstances in rural areas that the research team should be aware of.

#### Where to conduct the interview?

In terms of venue, the participants suggested conducting the interview at the family’s residence for confidentiality and convenience. Other options could include the village head’s house or the village meeting hall, where public gatherings usually take place, the monastery or pagoda compound, health centres or elsewhere where the respondent feels comfortable providing the information. However, on a unique occasion, like when someone was murdered, conducting an interview at home might not be safe due to potential harm to the participant. In such cases, the interview should be conducted at the village chief’s house, especially if there are lawsuits going on between the families. One Lao village representative reported,

If the families don't mind, then it can be done at their homes, depending on their preparedness. As a village representative, I don't mind meeting at the village meeting hall or at the family’s house. However, some families are different, and sometimes they don't want you to visit their houses; they want to meet at the village meeting hall instead. In my community, some village chiefs do not also like to visit the houses, and there is a spacious area in the village meeting hall for all concerns.

#### Who should conduct the interview?

Regarding gender preference for the interviewers, in some cultures, women are not freely permitted to communicate in private with men who are strangers, regardless of the subject matter, and traditional and gender norms are still internalised and adhered to. In our study, Akha and Lisu respondents mentioned,

Our community has a gender preference. If the respondent is a woman, it’s better to have a woman interviewer, as we feel more comfortable answering the gender-specific questions.

Similarly, the Bangladeshi FGD participants also expressed a preference for a male interviewing a male respondent, and a woman-to-woman approach. S’gaw Karen and Burman respondents had a mixed opinion,

To talk openly, the same gender, female-to-female, or male-to-male, sharing is great. For some people, as far as I can remember, a male interviewee is all fine, but if a female staff is present, it’s better.

The discussants in other FGDs expressed no strong gender preference, and opined that anyone was allowed to have the interviews with their families’ approval. Additionally, an Akha (one of the several ethnic groups present in northern Thailand) person said that an interpreter or a person who can understand the local dialect (Pasa Nuea) was especially favoured; otherwise, the research team might find it hard to interview, and interviewing about someone’s death is sensitive, as this has not happened before in this area.

#### How to conduct the interview?

There are likely to be uncomfortable circumstances or some challenging situations that may arise throughout the interview and appropriate management was recommended by the participants.

##### Preventing potential harm and emotional distress

The FGD participants expressed that VA interviewees could suffer from negative emotions (annoyance, frustration, sorrow or anger) during the interview, especially if there had been a very close relationship to the deceased. The family members may still miss, pray for and cry for the deceased person, even though he or she died some years ago. One Khmer woman who lost her baby a couple of years ago indicated,

I have lost my own child, and if I am interviewed about my child, it will become a new suffering for me immediately, and I feel a new grief.

In this scenario, the team should provide more time to the respondents and keep in mind that visiting the family is to share or relieve their grief, not to worsen it. If there is any emotional distress, the interview should be paused to calm the respondent. If the respondent still feels sad, it will be better to make a second appointment to conduct the interview the next day or wait until the interviewee feels ready to participate.

One Burman respondent who lost his beloved one still felt sad during the FGD activity and said,

When the person died, we did not want to keep any remembrance, like photographs or things. If we keep them, and when we see them again, the sad feeling arises.

Thai participants added that sometimes the cause of death itself caused emotional distress to the family,

It depends on the cause of death. If it is due to an accident or anything that crushed the hearts of relatives, the family might not be able to cope with some questions. If the questions are too detailed and they don't want to answer, they will say, “I don't know”.

A crucial point made during the Bangladeshi FGD is that an interviewer should assess the mental state of the respondents while obtaining informed consent, and the interview procedures should be done very politely and gently with sympathy. The implication is that an individual interviewer’s competence and conduct matters greatly.

##### Confidentiality of information

The protection of the confidentiality and privacy of the data is paramount during the interview and in the storing and sharing personal information. Most participants from Bangladesh reported that sometimes the community might avoid providing information in cases of unnatural or mysterious deaths. Similarly, one Thai participant spoke of keeping confidential information depending on the cause of death.

On this point, I have encountered such a problem before. One person of my relatives was murdered, and reporters interviewed me. I had to be very careful with my words because whatever I say will be magnified, and I will not be safe.

In this case, it was suggested that support from the village representatives or local authorities could be needed to assure the family that any information they provide will be treated as confidential.

The expression about maintaining confidentiality about a death with a stigmatised disease condition was made by a S’gaw Karen,

In my opinion, if a person dies of HIV/AIDS (there have been 1–2 cases in the past), talking about him will not affect him as he has already died. However, we should be mindful and consider the remaining family’s feelings. Also, we are not sure if the disease is transmitted to a family member or not. If we are not careful with our words, the family will face discrimination from society. It’s not that we are hiding this, but we need to protect the family’s reputation.

##### Increasing interview acceptance

Across all FGDs, participants said that interview refusals would not happen often, provided that the community was willing to participate and understood the purpose of the interviews. One Lao respondent said,

In the past, there has never been a time that my villagers refused to cooperate. They will answer as much as they can during the interview. Even in cases of refusal, the village chief will persuade them to cooperate, and most of the time, the issues are solved.

In addition, participants from Bangladesh said that in case of interview refusal, the team should get help from the religious leaders or village heads, as they are trustworthy to the local people. Alternatives include help from the local administration and from close relatives or neighbours to convince them.

Similar opinions were expressed by Burman, as one participant stated,

To avoid the interview refusal, if the team should include head of the ten or hundred households or section leader, winning is on your side and things will be fine.

Another Burman member said communication was key to encouraging the community, and she reported,

We will explain this study about the cause of death to the family, like if a person has a heart disease and dies suddenly, the cause can be due to heart disease, or there is a probability of other diseases that lead to death as well. So, in order to avoid cases like this, to prevent them, and to treat them, we have to explain and encourage them for the interview.

##### Other issues raised by participants

The participants were also keen to know the study findings about the causes of deaths in their village because many of them had remained undiagnosed or even untreated before death. One Lao village head emphasised,

I have been the village chief for many terms, and there has never been a study like this before. It’s important for us to take lessons or to know the facts from technical experts

A majority of participants reported that the aggregated results could be shared with the District Health Office, and they would be disseminated to the village chiefs. Other alternatives were via a team member or local health staff who can deliver the information. In general, providing feedback at the community level was seen as worthwhile if the deaths are mostly preventable.

Exceptional situations such as humanitarian crises, armed conflicts and violence are common in some of these areas, often involving large population movement and exerting extraordinary pressures on health systems. Mortality trends can, therefore, change dramatically over short periods, while vital statistics such as certification of deaths, notifications and filing are often declined at the same time. These challenging environments affect the feasibility of the VA activities and interpretation of findings. On the Thai-Myanmar border, there has been ongoing political conflict, which creates challenges to implementation. One Pwo-Karen residing on the Thai-Myanmar border mentioned,

There are a lot of people from other parts who come to this area, as the situation is not good. Due to the political conflict, at the military checkpoint when the research team used to enter, they would first have to explain, when asked, the purpose of commuting as we are working on health-related issues and diseases etc. to the authorities. Then, they have to go to see the administration and health worker groups to have the discussions and seek permission. If they permit us, we have to go to the village chief and health volunteers to talk about the study and procedures too.

## Discussion

In this qualitative study, we explored community stakeholders’ opinions of the ethnocultural and practical aspects for conducting VA interviews in five rural South and Southeast Asian settings. This activity aimed to improve research procedures and mitigate against potential harms to future study participants. We found that most participants perceived death as a part of human nature while recognising the importance of community support for the family with physical, financial and spiritual aid. In all these communities, religious and village leaders play an important role in village life and especially at times of death. Rituals surrounding death are seen as essential, and families would be upset if there were any intrusions and they would not able to correctly perform the rituals for their loved ones, to prepare them for their next life, rebirth or reincarnation. Yet discussions revealed that these societies are not static, and some traditions change over time. In the Hmong community of Thailand and S’gaw Karen community, some elderly people still worship their ancestors, but the younger generation nowadays do not perform many ritual practices, like the sacrificing pigs and buffalos.

Despite the importance of interview timing, the evidence available in the literature is limited and not easily generalisable from one setting to another. One study proposed that between 6 days and 5 months after the death was optimal,^13^ and another study reported that this varies from 100 days to even 1 year after the funeral.^14^ The participants in our discussions also provided no generalisable instructions as to how soon after death the VA should be conducted (ranging from 3 days to 100 days), but they did inform us that this should be approached on a site-by-site basis, and that for deaths of a more sensitive nature, even a case-by-case approach should be considered. While the VA interview may cause potential emotional harm to participants, this can be largely avoided with proper communication and procedures by using suitable words and manners and paying respect to community traditions and customs. In addition, respondents reported it was necessary for the interviewers to be accompanied by a local trusted person, make contact in advance, have interviews appropriately scheduled and clearly explain the purpose of the interview in order to facilitate cooperation. The venue of the interview should also be considered as important, as it influences data collection.^15^ We were recommended interviews be conducted at a place of the respondents’ convenience, in a comfortable setting, and free from any potential interruptions. We were also advised that it is important to share the grief with the families and show empathy during the interview. Of note, the opportunity to talk about the death of a loved one may be welcomed by members of some communities. This perspective has been observed previously in Vietnam and Malaysia.^14 16^ It was also suggested by a Thai respondent to frame the interview as a social visit, and to promote ethical practice, the team can bring some goods or small gifts, like water or snacks (they do not need to be valuable); if the family seems to be struggling financially, providing some compensation is a fair balance of benefits and inconvenience for the families. A Nepali study has also reported these same practices.^9^

A body of research has explored whether the characteristics of interviewers, particularly gender and ethnicity, affect the responses to interview questions^17 18^ and the interviewer-effects in public health surveys are of growing interest.^19 20^ Our study revealed a preference for female interviewers for female respondents to speak about female-specific health topics, and also a preference of same ethnicity who can speak local dialect among some ethnic groups. A qualitative study conducted in India^21^ concluded that the consideration of interviewer gender was important in designing survey protocols for reproductive health-related studies or sensitive topics. Therefore, in VA studies too, sufficient numbers of women should be trained as interviewers. Public opinion research in Bangladesh,^22^ the USA^20^ and among ethnic minorities in Europe^23^ also report ethnicity-of-interviewer effects, and when interviewed by a member of their own ethnicity, participants may express significantly different views. Therefore, the translation of tools and the correct selection of local interviewers who understand local terminology and language are essential for putting respondents at ease and collecting accurate narratives, especially in our VA study where multiple ethnicities may be encountered even within a single site.

Maintaining confidentiality is important when documenting deaths in small rural communities and is critical to protect participants, particularly in areas of armed conflict and distrust of outsiders or authorities such as at the study site in Myanmar. Another important bioethical concern around VA is the awareness of potential emotional distress for both the interviewers and interviewees. The respondents sometimes feel offended and distressed when they are asked about their deceased family members. On the other hand, the interviewers would steadily witness and experience the pain, suffering and unhappy moments of research participants during their daily research work.^24^ The focus of the present study was only on communities’ perceptions and did not investigate any potential distress of the research team or interviewers. There is a paucity of studies that have sought to elicit data collectors’ views on their experiences.^25 26^

Further qualitative research to uncover the viewpoints of communities and government authorities could improve the current death notification and registration. In LMICs, under-reporting of deaths is currently common due to lack of legal enforcement; deaths are often not formally reported or registered by the family for reasons of distance and economic and cultural restrictions. This is further exacerbated by delays in the information being transferred from the death registration office to the vital statistics system, particularly when forensic and police investigation is involved. Stillbirths and neonatal deaths are also commonly under-reported due to a lack of awareness in the communities.^27^ A better national mortality statistics could be achieved by putting more emphasis on these issues.

A summary of recommendations from these FGDs provides location-specific guidance for the main VA study (table 2), which is currently being conducted at the five sites. This includes how the researchers should deal with families when an unnatural death has occurred, how to have a smooth interview process with the communities in a kind, polite and convincing way, suggestions for dealing with the emotional distress of participants and approaches to increase the response rate and confidentiality. The implementation of the VA study at the study sites might become a concern, as face-to-face interview is a standard method for the VA study, while COVID-19 remains a regional public health concern. Furthermore, the benefits and demands of research should be carefully considered in one of the study areas, where armed conflict and violence are evolving.^28^ Acknowledging the operational and organisational challenges, timely data collection using standardised tools and community involvement were important factors for the successful study implementation.^29^ The systematic use of FGDs at each site provided necessary knowledge to initiate the VA study at ethnoculturally diverse locations across the region. The communities’ leaders also demonstrated their interest in participating which gave confidence to the research teams.

**Table 2:** Recommended approach for verbal autopsy

Concerns	Recommendations by FGD participants	Justifications
Interview timing	Carry out interviews after the mourning period, following local practices; and also reconsider on a case-by-case basis.	Providing appropriate time for the respondent to get ready to prevent potential emotional distress and also be aware of recall bias
Emotional distress of the participants	Assess the emotional status of the respondents while obtaining informed consent. Pause the interview and divert the respondents’ attention with other conversation if emotional distress occurs. If the conversation cannot be continued, try to reschedule a second interview. If the second interview does not work, find another option such as asking another close relative or community leader or neighbours instead (only if they have the required knowledge to accurately answer the questions)	Address the participants’ distress and provide mental health support if needed
Unnatural cause of death or natural deaths due to stigmatised communicable disease	Clearly explain the purpose of the interview to the respondents, ensure the information is kept confidential and avoid making, or give the perception of making, any moral judgement.	Recognise the stigmatising attitudes of others and support the family by respecting their private information
Interview refusal	Get support from a local authority or village representative who can encourage the families to cooperate.	Comply with research ethics (if the family is still hesitant to cooperate for any reason, respect the family’s decision)
Dissemination of the study findings	The findings should be disseminated to the communities as well as all relevant stakeholders through official channels.	Ensuring both the study community and all stakeholders benefit from the research.

FGD, focus group discussion.

Having a heterogeneous group in our study revealed diverse perspectives on how the community perceives deaths and appropriate practices for the VA study. We view this diversity as a strength providing richer insights than might have been obtained with a more homogenous selection of participants and this approach made a very practical option for us with constrained time and financial resources. On the other hand, our study’s limited generalisability to the entire population stems from the fact that it was particularly conducted based on designated locations (where the specific population resided) of future VA study, which is one of the study’s shortcomings. In addition, we were unable to thoroughly address the potential biases in our study–participant’s and researcher’s bias. However, we made every effort to minimise it through a variety of means, including developing open-ended research questions, conducting the FGDs expertly and having the transcribed data checked by the local study teams, the data analysed by experienced researchers and the findings reviewed by a senior researcher/mentor for a second, unbiased opinion. Analysing the qualitative data derived from between and within site variations and reporting the findings is quite challenging. However, we provide a succinct summary of the findings, including one or two notable quotes for each main theme and subtheme. We were unable to cite every statement made by all FGD participants due to space restrictions and a variety of viewpoints.

This study was undertaken with the goal of successfully conducting the VA study while taking into account local cultural traditions and sensitivities towards VA procedures. Although ethnocultural beliefs vary between the different locations where VA is done, the topics we have identified in this study may provide guidance to interview teams in other such studies as to the issues to be taken into consideration and how these can be approached in advance. Documenting the process of learning about local cultural norms prior to initiating VA studies has not been reported widely, and, therefore, the FGD’s findings will enrich the literature on the VA research.

## Conclusion

In the absence of a complete vital registration system, LMICs can improve estimates of causes of death by verbal autopsies. We found that VA is acceptable across a wide range of cultural settings in rural South and Southeast Asia, provided that local norms are pre-identified and followed.

## Data Availability

All data relevant to the study are included in the article.
