# Supplementary material for: Ethical and cultural implications for conducting verbal autopsies in South and Southeast Asia: a qualitative study
Source: BMJ Glob Health. 2023 Dec 11;8(12):e013462. doi: 10.1136/bmjgh-2023-013462 (PMC10729118; doi:10.1136/bmjgh-2023-013462)
Supplement: Supplementary data [file bmjgh-2023-013462supp002.pdf]

Supplementary Appendix 2 – Author reflexivity statement

1. How does this study address local research and policy priorities?

This author reflexibility statement is specifically designed to address research equity within international partnerships. This is an issue of major priority in low- and middle-income countries whose researchers engage in collaboration with high-income country researchers. This study was done to understand cultural sensitivities around verbal autopsy, a method used to understand causes of death in areas with limited death registration. Improving the quality of verbal autopsy practice can contribute to better and more reliable local research. While the better understanding of causes of death among the rural poor can improve health policy by informing the better targeting of limited health resources.

2. How were local researchers involved in study design?

This study was designed and led by a female medical doctor from Myanmar working as a post-doctoral researcher at a University in a LMIC. The first category of local researchers involved were those with extensive experience of involvement conducting, leading, or organising international research collaborations (NSNH, RC, NK, PYC, TJP and YL in Thailand). The second category was local researchers with ongoing experience with management of field activities at the ground (APP in Thai-Myanmar border, CP in Thailand, MV in Cambodia, KP in Lao PDR, AS in Bangladesh). There were also researchers who are currently based in high-income countries, but with experience of working as local researchers within international research collaborations (SKM and PJ in Canada, AO in UK). In our manuscript, the first and second category of local researchers who act as researchers as well as journal editors, took the lead in developing study design.

3. How has funding been used to support the local research team?

This project has been used to leverage approximately £10,000 to support a post-doctoral researcher based in Thailand (NSNH) to develop the study design, implementation, data analysis and to produce a manuscript.

4. How are research staff who conducted data collection acknowledged?

All research staff who got involved in this FGD activities across the sites were included as authors.

5. Do all members of the research partnership have access to study data?

All members of the partnership have access to data.

6. How was data used to develop analytical skills within the partnership?

The data were translated from local languages to English and verified by the local researchers who carried out the FGDs. The data analysis was carried out by NSNH in collaboration with the local researchers at each site. Then, all of the authors—from high- to low- and middle-income nations—with multidisciplinary research backgrounds offered their opinion on the findings. Therefore, all members of the partnership have gained analytical skills through this work.

7. How have research partners collaborated in interpreting study data?

During the development of the manuscript, the first author had meetings with the research sites to clarify the manuscript's contents and language and prevent misrepresentation. All study partners received the manuscript's initial draft in order to provide technical comments and enhance the manuscript's quality. The final draft was adjusted in response to comments from all partners; no conflicts were reported; a flexible statement of authorships for researchers from high- to low- and middle-income nations was agreed upon; and everyone provided an approval for submission to the journal.

8. How were research partners supported to develop writing skills?

The research team writing and leading this manuscript were pre- and post-doctoral early career researchers (NSNH, CP, KP, APP, RT, RC, AO) on the authorship team were supported by senior academics within their working groups to develop and refine their writing skills.

9. How will research products be shared to address local needs?

This manuscript will be published as open access. There will be a dissemination session to share the findings especially with the verbal autopsy study teams and relevant stakeholders across the sites.

10. How is the leadership, contribution and ownership of this work by LMIC researchers recognised within the authorship?

Authors NSNH, YL and TJP are the leading authors (who are based in Thailand) in developing this manuscript, and their contribution has been recognised as first and joint last authors respectively.

11. **How have early career researchers across the partnership been included within the authorship team?**

We have included early career researchers (NSNH, KP, APP, AO) within the authorship team. They contributed to the data synthesis and to development of the manuscript.

12. How has gender balance been addressed within the authorship?

Eleven authors are male (CP, APP, AS, KP, WY, RT, RC, SKM, PJ, YL, TJP) and twelve authors are female (NSNH, MV, NK, JS, JW, NK, XX, AS, KS, NK, AO, PYC).

13. How has the project contributed to training of LMIC researchers?

Chiefly, the study lead and first author is a post-doctoral researcher from Myanmar, who has developed her qualitative research skills through this study. This work was done in collaboration with early career researchers in each study country (Bangladesh, Myanmar, Thailand, Lao PDR and Cambodia).

14. How has the project contributed to improvements in local infrastructure?

This project has not directly contributed to improvements in local infrastructure.

15. What safeguarding procedures were used to protect local study participants and researchers?

We have specifically considered the issue of safeguarding within the manuscript. The study's deidentified data ensures that participants' identities cannot be discovered.
