# Supplementary material for: Ethical and cultural implications for conducting verbal autopsies in South and Southeast Asia: a qualitative study
Source: BMJ Glob Health. 2023 Dec 11;8(12):e013462. doi: 10.1136/bmjgh-2023-013462 (PMC10729118; doi:10.1136/bmjgh-2023-013462)
Supplement: Supplementary data [file bmjgh-2023-013462supp001.pdf]

Supplementary Appendix 1

Appendix 1a: Bangladesh

Main religions and ethnic groups	The study sites are Cox's Bazar and Bandarban districts on the southeast coast of Bangladesh. Muslims (Bengali) are the majority, and a few are Buddhists and Hindus. Marma is one of the ethnicities in Bangladesh, and they are Buddhists.
Funeral practices	In Bangladesh, the dead person is first bathed with water, placed on a bamboo platform called "matcha" (used by Marma) or "Pati" in Bengali, then covered nicely with pieces of clothing (5 pieces for males and 3 for females) and carry the body in a wooden coffin (called "khatia") to the graveyard for burial. The dead person's relatives, neighbors, and religious leaders (monks in Buddhist communities who perform "punnodan") pray for him or her at home, or mosque, or at the graveyard (in the Muslim community). Buddhists and Hindus in Bangladesh either bury or cremate the body which usually depends on the wish of the deceased except the children who are usually buried. However, a person who dies by hanging or poisoning is cremated. Between the 5th and 7th days, feeding underprivileged people is performed, believing that the deceased can get more virtue in the afterlife. The "Somprodan" occasion is held for 15 days, 1 month, 6 months, and also 1 year, according to the economic capabilities of the family.
Mourning period	Hindu: 7 to 40 days; Buddhists: mostly 7 days and can be up to 1 year; Muslims: 4 days up to 6 months
Traditions and taboos	Interestingly, Marma (another ethnic group) shared that after the death of an elderly person, grandchildren dance and sing with a festive mood, carrying the dead body to the graveyard for completing rituals and celebrating long life, and that in their society, females can go to the graveyard while other religions don't permit females to go.
Particular sensitivities or concerns to VA study	The team should be aware of possibility of getting information related to some unnatural deaths. In the case of an unnatural death, if the true information is revealed, it may cause harm to them.
Neonatal and child deaths	Grief time really lasted for a long time for a mother when the first child died (shared by a Hindu mother).
Unnatural deaths	The team might face difficulties capturing unnatural deaths like poisoning, hanging or any mysterious cases.
Implications for approaching participants	The team should inform the village authorities and the interview arrangement should be done through them.
Implications for providing compensation	Not mentioned
Implications for interview time and location	The interview should be conducted 1-3 months after the natural death occurs and wait for a longer period for unnatural deaths. The interview can be done at any comfortable places.
Implications for who does the interviews	Interviewing man by a man, and woman-to-woman is preferred.
Implications for getting back the study results	The community will be happy to hear the diagnosis of their beloved ones who passed away, as many of them even had remained undiagnosed or even untreated before death.

Appendix 1b: Cambodia

Htun NSN, *et al. BMJ Glob Health* 2023; 8:e013462. doi: 10.1136/bmjgh-2023-013462

Main religions and ethnic groups	The study sites are Pailin province and Samlout, Koas Krala and Rukh Kiri districts of Battambang province in the far northwest of the Cambodia. The majority are Khmer Buddhists.
Funeral practices	In Khmer tradition, when a person dies, the Achar (local religious elder) uses a preservative substance to preserve the corpse from smelling and decaying, and then the corpse needs to be cremated within 3 days. The villagers invite the Buddhist monks for the blessing, and help the families with cooking for guests. They also help raise contributions to support all suffering families, especially the poor. The community can contribute based on their kindness in the form of money (one thousand, twenty thousand, forty thousand riels, etc.) or things for cooking (rice, vegetables, sauce, etc.). In some villages, there is an association called "the Association for Dead People" to help the families prepare for the ceremony.
Mourning period	The duration of mourning depends on the available resources and usually lasts between 2-3 days, but mostly 7 days. Some families mourn for a longer time, at least 5–6 months. Elderly people can be compared to 'ripe fruits', and when they pass away, the family does not mourn for long. However, for young people who are just 18 years old or younger, Khmer people mourn their deaths for a longer period of time.
Traditions and taboos	For mourning purposes, the family members use a small piece of fabric to be fixed to their shirt for three days or one week. There are no taboos about deaths when the participants express them in the communities, believing that it was the people's right to talk about that, and they are no longer afraid of corpses because the corpses are cremated.
Particular sensitivities or concerns to VA study	There are no particular concerns about the VA study, as the purpose of the study is to find out the causes of the illness and death.
Neonatal and child deaths	For a mother who lost a child in an unexpected tragedy, it is hard to forget her dead child, and the interview should be conducted after a careful consideration.
Unnatural deaths	Those who died in traffic accidents are the most serious remembrance for the family members due to a sudden onset and the family usually cannot cope well after death.
Implications for approaching participants	If the team wants to visit the house, the community or village authorities should be contacted, and then an appointment could be made after checking the availability of the respondents, because sometimes s/he might have gone to his or her farm and not present at their residence.
Implications for providing compensation	Some local products to use for the family would be fine.
Implications for interview time and location	If necessary, interviewers are welcome to do the interviews, even during the mourning period or one week after the ceremony. Some suggested to wait for the interview for a month and a half. The sorrow can also be longer for a mother, father, or child, as they keep struggling with the grief over the loss.
Implications for who does the interviews and how	The questions should be precise and short and only focus on the history of sickness. The interviewers should not make the mourners more sorrowful. The interviewers should be gentle and polite with their wording. The community has no gender preference for the interviewers.
Implications for getting back the study results	Not mentioned.

Appendix 1c: Lao PDR

Htun NSN, *et al. BMJ Glob Health* 2023; 8:e013462. doi: 10.1136/bmjgh-2023-013462

Main religions and ethnic groups	The study sites are Atsaphangthong, Phine and Phalanxay districts of Savvanakhet province located in Southern Laos. The majority of Lao are Buddhists, followed by animist and a few are Muslims, Christians, and Hindus.
Funeral practices	In Lao community, when a person dies, the body is washed as a cleansing ritual and then placed into a casket for a wake. During a Laos wake, friends and family are welcomed to pay their respects and this is a time for supporting the grieving family by bringing money, flowers, food. If the person dies unexpectedly or in an accident, the body is to be kept in the temple for at least a day before the funeral. When convenient the monk is invited to bless the copse in the morning or at night. The Lao community accept both burial and cremation though it’s more common for people to choose cremation and believe in reincarnation — the cycle of death and rebirth.
Mourning period	After the funeral, the deceased's family has several memorial services to honor their memory, especially 100 days and one year after the death. Phouthai ethnic group has a 49-day mourning ceremony.
Traditions and taboos	No traditional restrictions or taboos is mentioned.
Particular sensitivities or concerns to VA study	In the harvest season, the family members are occupied in the field and are not at home, especially in the afternoon. Sometimes, the family still feels sad about their loss and do not want to answer the questions well; in this case, the team should reschedule the interview. One village head mentioned that he was unaware of the VA study until FGD began, and no one briefed them on what the project was about. The team was also suggested to prepare an official request letter for them (with district or provincial authorities’ approval) showing the legitimacy of the study or organization, which would allow the team to visit the family.<br>Emotional distress: If the respondents are sad or start to cry, the interviewer should not continue with the questions but let them calm down, then continue, or the interview can be rescheduled.
Neonatal and child deaths	No particular thoughts have been mentioned
Unnatural deaths	The cause of all deaths, including unnatural deaths (e.g., accidental deaths), has never been kept secret in the Lao communities.
Implications for approaching participants	The team or interviewer has to come to the village chief or authority, and then the village representative will accommodate him/her with the family for the interview. Sometimes, family members or relatives can’t give the information, but the village authorities or some people who are close to the deceased person/s can provide it.
Implications for providing compensation	Some useful products for the family sound reasonable for sharing grief and kindness.
Implications for interview time and location	The villages usually have a tribute fund, which is collected once the death is known. The tribute fund is handed over to the family at the funeral or at the house warming, and the interview can be conducted at the house at that time, as the village head is also be present. This ensures family members are at home and available to provide information. However, if the interviewer comes and visits the family alone, the family will not cooperate for sure.<br>Another possible time is after the housewarming ceremony and funeral, as the interviewer can visit right away without waiting 2-3 months, or anytime upon the family’s approval.<br>In terms of location, at family’s homes or village meeting hall is fine in Khmer villages.
Implications for who does the interviews	No gender preferences

Htun NSN, *et al. BMJ Glob Health* 2023; 8:e013462. doi: 10.1136/bmjgh-2023-013462

Implications for getting back the study results	The participants believe that it is important for them to take lessons learned from technical expert.

Appendix 1d: Myanmar (Thai-Myanmar border area)

Main religions and ethnic groups	The study sites are Hpa-An and Myawaddy districts of Karen state on Myanmar-Thai border. The majority are Karen (Christians), and a few are Burmans also known as Bamars (Buddhists)
Funeral practices	In the Buddhist community, when a person dies, most likely there will be a one-day funeral, followed by a seven-day memorial. Before the deceased's body gets stiff, it has to be washed, dressed properly, and put in the coffin. The body is then accompanied by all the relatives to be cremated. The monks then perform the funeral service rituals (prayers and chants), and family members pour the water into the ground, a standard part of Buddhist spiritual discipline to transfer the merits to the deceased. Cremation is done afterwards. Then people head home to clean their hands and feet (or whole body) with a holy water with soap nuts and turmeric. During memorial days, the villagers come visit and sing songs at the deceased's house until the period passes. Nowadays, the current generation only practice this using a cassette tape, not a person. After one week, the monks are invited to home to chant, a meal is offered, and at that time, the family will invite the spirit of the dead one to come home to receive the merits done by the family. The villagers go to help the families during this time of grief. However, in Buddhism, if a person dies during a lent period, the death body can be sent to the cemetery, but funeral ceremony or any religious activity cannot be performed, and the family need to wait 3 months. The Buddhists believe to do good things as much as they can, such as meditation or not drinking alcohol during this period. The monks should stay in a monastery without traveling for 3 months.<br>For Christians, when someone dies, they are buried the next day, and the funeral reception lasts for two weeks.<br>In the Pwo-Karen community, it is different how to treat the dead bodies of adults, children, and unnatural dead. The new-born and children aged under 10 years are mostly buried. Adults are usually cremated, but in cases of unnatural deaths (motorbike accidents, car accidents, etc.), the body is buried as soon as possible. For religious leaders (pastors or monks) and rich societies, formalin is injected into the body to prevent the decaying process, and memorial service also takes longer (e.g. for a year).<br>There are some elders who are animists and there is a ritual with animals, e.g. when an animist passed away, people brought his chicken for cremation. His body and alive chickens along with the basketwork (to keep the chickens in) were thrown into the fire together. However, his children/younger generation do not continue this animism practice anymore.
Mourning period	It varies among the families, ranging from 3 days to 2 months, depending on how close the deceased person was with the family members.
Traditions and taboos	If a person dies somewhere outside of the village, the villagers think that the unnatural deaths would cause the spirits to haunt them as well as it brings back luck; therefore, those corpses were not allowed to bring back into the village. However, if the headman allows to do so, one pack of salt should be kept at the head

Htun NSN, *et al. BMJ Glob Health* 2023; 8:e013462. doi: 10.1136/bmjgh-2023-013462

	man's house or at junction of the road, and the villagers need to go and pick small amount salt and spread it under their house or eat it or hang the salt pack on the door.<br>Another belief is that the people who send off the dead body to the cemetery cannot look back or cannot hold each other's hands. After sending off, the people need to wash themselves (the whole body) with holy water, which is made out of soap nuts and turmeric to clean off the bad things. If they do not do so, the children might get sick.
Particular sensitivities or concerns to VA study	Recently, there have been a lot of people who immigrated or emigrated from the area due to the unstable political situation. The research team might face some difficulties following up on the cases and conducting interviews with the families.
Neonatal and child deaths	If a young child under 1-year dies, they are usually buried on the same day. When a pregnant woman dies, her womb is cut to take out the baby, and then, the mother and the baby are buried separately. A mother can be emotionally traumatized for a long time if her child died of unexpected circumstances.
Unnatural deaths	In the case of a stigmatized disease like HIV, the family does not like to disclose why and how he/she died. The family feels uncomfortable, and it would be a disgrace to them if society knew the cause of the death.
Implications for approaching participants	Visiting the families with the village leaders or section leaders (in a larger village) is most appropriate.
Implications for providing compensation	Not mentioned
Implications for interview time and location	The interviews should be conducted 3 or 7 days after the memorial and the family's house is most suggested for the interview location.
Implications for who does the interviews	The respondents thought that the community might prefer a same-gender approach for the interviews (woman-to-woman), as some women might feel uncomfortable when talking with men.
Implications for getting back the study results	The community would like to know the result of the study, and it should be done through village authorities.

Appendix 1e: Thailand

Main religions and ethnic groups	The study sites are Chiang Khong, Khun Tan, Mae Suai and Wiang Kaen districts of Chiang Rai province of Northern Thailand. Most of the Tai Yai, Yunnan Chinese, Khmu, Hmong, and Lao-Thai are Buddhists, and the Lisu, Akha, and Lahu ethnic groups are mostly Christians.
Funeral practices	In Buddhist tradition, when a person dies, the body is immediately washed, dressed, placed into a coffin, and kept in the house for three to seven days before cremation or burial. The monks are invited to home or (to the monastery in the Thai community) to recite a sermon or to chant for the blessing. After the body is cremated, the ashes are collected (spread into the water in some cases) or, if buried, a small house archway with a pagoda or stupa is made within the temple compound. If the deceased is a Christian, he/she is mostly buried and rarely cremated. Religious leaders are invited to pray and read the Bible at the ceremony. Then the family holds the funeral ceremony, depending on their socio-economic status.
Mourning period	Usually about one week in Thailand, regardless of religion.
Traditions and taboos	In the Khmu community, if someone is killed, the body is only kept for a short duration (2 days) before being cremated, believing that it would bring disaster or bad luck to the family. Families also worry that it may be

	difficult to send the souls to reincarnate. In the tradition of the Lisu ethnicity, some pieces of silver that are similar to pieces of milled rice are put in the mouth of the deceased: 9 pieces of silver for a man and 7 pieces for a woman. This is only done to elders and is not applied to children or adolescents.<br>When a member of the Akha ethnicity (most are Christians but, in the past, they used to be animists) dies, the fingers of the deceased are bound together with white thread, the body is bathed, and wrapped in black fabric. If children or adolescents never got married, white fabric is used to wrap them. After the corpse is wrapped up, it is laid next to the door in order to perform the funeral ceremony, and religious leaders are invited to pray. Most of them keep the corpse for 3 days, depending on the family resources; the family must slaughter 1 pig per day (i.e., 7 pigs to keep for 7 days).<br>When a member of the Lahu ethnicity dies, there is a gun shooting into the sky to give a signal that someone has just died. On the day when the body is cremated, everyone stops working, believing that if they go to work, the deceased will not be able to eat what is offered.<br>In the Hmong community, for 3 years after the death of parents or grandparents, it is forbidden to kill wild and domestic animals, and also to dispute certain issues, believing that the family might not advance in what they are aiming to do. Also, if a married woman dies at her parent’s house, she is considered an outsider, and the ritual is done at her husband’s family house. In the Thai community, it's very important to address the debt of the deceased person, assuming that if there is a debt, he or she can’t reincarnate.<br>There is no taboo talking about deaths in the community.
Particular sensitivities or concerns to VA study	In the Lisu community, it is prohibited to take any photos in the house in order to respect the ancestors. If the interviewers are willing to do so, permission must be sought from the household heads.<br>Sometimes, if the first respondent is in emotional distress (parents, spouse), it is best to ask the community’s leaders or a relative or close friend instead. The team may get better answers that are also less heartbreaking for the family members.
Neonatal and child deaths	The mother can still be heartbroken with her child’s death. In this case, close relatives or friends could be interviewed alternatively, and they do not need to be the parents.
Unnatural deaths	The community/family might not like to take the interviews for some specific conditions like communicable disease (HIV), murder, or conflict.
Implications for approaching participants	It is recommended to approach the community through community leaders, village chief, primary care unit staffs or village health volunteers.
Implications for providing compensation	According to tradition, small gifts or consolation goods, water, snacks, or other items are adequate.
Implications for interview time and location	It can be after the funeral ceremony, or between 1 and 3 months, but it depends on the case. If the approach implies mental support for the family, they would welcome the interviewer.<br>The venue of the interview could be either at the family’s house or the house of the village chief.
Implications for who does the interviews	The Lisu community prefers a woman interviewer to interview a woman respondent.
Implications for getting back the study results	Not mentioned
